# Effects of Carpal Tunnel Syndrome on Adaptation of Multi-Digit Forces to Object Weight for Whole-Hand Manipulation

**DOI:** 10.1371/journal.pone.0027715

**Published:** 2011-11-16

**Authors:** Wei Zhang, Jamie A. Johnston, Mark A. Ross, Anthony A. Smith, Brandon J. Coakley, Elizabeth A. Gleason, Amylou C. Dueck, Marco Santello

**Affiliations:** 1 School of Biological and Health Systems Engineering, Arizona State University, Tempe, Arizona, United States of America; 2 Faculty of Kinesiology, University of Calgary, Calgary, Alberta, Canada; 3 Mayo Clinic Hospital, Phoenix, Arizona, United States of America; The University of Western Ontario, Canada

## Abstract

The delicate tuning of digit forces to object properties can be disrupted by a number of neurological and musculoskeletal diseases. One such condition is Carpal Tunnel Syndrome (CTS), a compression neuropathy of the median nerve that causes sensory and motor deficits in a subset of digits in the hand. Whereas the effects of CTS on median nerve physiology are well understood, the extent to which it affects whole-hand manipulation remains to be addressed. CTS affects only the lateral three and a half digits, which raises the question of how the central nervous system integrates sensory feedback from affected *and* unaffected digits to plan and execute whole-hand object manipulation. We addressed this question by asking CTS patients and healthy controls to grasp, lift, and hold a grip device (445, 545, or 745 g) for several consecutive trials. We found that CTS patients were able to successfully adapt grip force to object weight. However, multi-digit force coordination in patients was characterized by lower discrimination of force modulation to lighter object weights, higher across-trial digit force variability, the consistent use of excessively large digit forces across consecutive trials, and a lower ability to minimize net moments on the object. Importantly, the mechanical requirement of attaining equilibrium of forces and torques caused CTS patients to exert excessive forces at both CTS-affected digits and digits with intact sensorimotor capabilities. These findings suggest that CTS-induced deficits in tactile sensitivity interfere with the formation of accurate sensorimotor memories of previous manipulations. Consequently, CTS patients use compensatory strategies to maximize grasp stability at the expense of exerting consistently larger multi-digit forces than controls. These behavioral deficits might be particularly detrimental for tasks that require fine regulation of fingertip forces for manipulating light or fragile objects.

## Introduction

Skilled manipulatory behaviors require complex spatial and temporal coordination of the digits that can be flexibly adapted to object properties such as size, friction, and weight. In healthy individuals, visual and somatosensory feedback is processed and integrated with motor commands to control multiple digit forces [1]–[5]. However, the delicate tuning of digit forces to object properties can be disrupted by a number of neurological and musculoskeletal diseases. One of the most common and debilitating conditions affecting hand function is Carpal Tunnel Syndrome (CTS).

CTS is a compression neuropathy of the median nerve resulting in sensorimotor impairments in the hand that begin with deficits in sensation in the thumb, index, middle, and lateral half of the ring finger (palmar and the most distal dorsal aspect of these digits) and progresses, in severe cases, to include motor deficits predominantly in the thumb. Symptoms include aching and burning, tingling, numbness, weakness, and clumsiness in the affected hand. The median nerve is a mixed nerve comprised of both sensory and motor axons innervating most extrinsic hand flexor muscles and some intrinsic muscles. It also relays sensory information from the palmar aspect of the thumb, index, middle and the lateral half of the ring finger. Prolonged mechanical compression of the nerve results in ischemic damage and/or changes in the myelination of the nerve leading to slowing of axonal conduction velocity, nerve block, and in severe cases axonal loss [6]–[7].

CTS affects several classes of sensory receptors required for grasp control, i.e., tactile mechanoreceptors of the glabrous skin as well as muscle, joint, and tendon receptors of intrinsic hand muscles. While the role played by mechanoreceptors of the glabrous skin for object grasping and manipulation has been extensively studied and established (see [8] for review), much less is known about the role of other classes of receptors in hand muscles, tendons and joints for grasp control. Throughout the manuscript we will use the term sensory deficits to denote damage to sensory axons belonging to the median nerve of all classes of receptors. Extrinsic digit flexor muscles are unaffected by CTS because innervation of these muscles occurs proximal to the site of nerve compression, whereas force production from intrinsic muscles including abductor pollicis brevis, opponens pollicis, flexor pollicis brevis, first and second lumbricals, can be impaired. Therefore, CTS may challenge the ability to coordinate intrinsic and extrinsic muscles acting on a digit, hence affect force coordination across digits [9]–[12].

The effects of CTS on nerve function are normally quantified by nerve conduction or electrodiagnostic tests consisting of electrical stimulation and recording the response either in the muscle it innervates or the nerve itself [13]–[18]. The response, which is compared with normative data based on the age and, for some measures, the gender of the patient, can yield useful information about the extent of axonal loss and demyelination. Other measures used to infer CTS-induced deficits of nerve function consist of testing the detection threshold of vibration, pressure, and light touch, as well as provocative tests, e.g., Phalen's and Tinel's tests. However, the extent to which electrodiagnostic and provocative tests predict hand function remains unclear and highly controversial [6], [19]–[21], [22]–[24]. For example, Cole et al. [20], in agreement with earlier observations [25], found that measures of light touch and grip force amplitude were poorly correlated with the degree of median nerve compression as quantified by sensory nerve action potential (SNAP) amplitudes. Furthermore, grip force and tactile sensitivity were differentially affected by the degree of nerve compression. These findings suggest that frequently used measures of tactile sensitivity are not strong predictors of nerve function, but also that inferences about grip force control for object manipulation cannot be made from either tactile sensitivity tests or measures of SNAP amplitude. This discrepancy between measures of nerve function and grip force control may be due not only to deficits in tactile sensitivity but also in using tactile input for planning digit forces. Specifically, feedback from tactile afferents contributes to the formation of sensorimotor memories that, in turn, enable subjects to predict object properties in an anticipatory fashion and plan grip forces accordingly [1], [26]–[28], for review see [8].

Despite impaired sensory function in digits, CTS patients are able to modulate grip force similar to controls in anticipation of load force to different frictional conditions between the object and digits [23]. This finding suggests an involvement of residual tactile sensitivity through afferent fibers spared by the median nerve compression. Yet, another study reported that CTS patients exert significantly larger forces than controls [29], thus suggesting a compensatory strategy to prevent object slip similar to that found in response to anesthesia of the fingertips [30]–[33]. However, the CTS studies have examined tasks involving the affected digits only, thus leaving unanswered the question of integration of tactile input across CTS-affected and -unaffected digits. Specifically, control of whole-hand grasping and manipulation poses the challenge to CTS patients of integrating sensory information with motor commands from multiple digits, a subset of which is characterized by deficits in sensorimotor capabilities. Most importantly, however, the mechanics of five-digit grasping may challenge CTS in different ways than two-digit grasping. In two-digit grasping, where both thumb and index finger exhibit sensorimotor deficits, excessive forces exerted by the two digits (see above) would not interfere significantly with object manipulation as long as they are collinear and of approximately equal magnitudes. In contrast, for five-digit grasping finger forces need to be distributed such that they do not generate torques while grasping or lifting the object to prevent the object from rolling. To attain this objective, feedback from the CTS-affected digits, *as well as non-affected digits* (part of the ring finger, little finger), has to be integrated for effective coordination of multiple digit forces. The extent to which CTS patients are able to coordinate multi-digit forces for grasping by integrating feedback from digits with impaired and intact sensorimotor capabilities remains to be addressed. Another gap in our understanding of the effects of CTS on multi-digit grasp control is the extent to which median nerve compression impacts trial-to-trial adaptation of multi-digit forces to object properties for skilled object manipulation.

The present study was designed to quantify trial-to-trial adaptation of multi-digit forces to object weight. We hypothesized that CTS patients would maintain the ability to modulate grip forces to object weight while using larger grip forces than controls with both CTS–affected and –unaffected digits. However, based on impaired median nerve function and expected deficits in tactile sensing, and the above evidence on the role of tactile sensing on the formation of sensorimotor memories we also hypothesized that CTS would affect trial-to-trial adaptation of multi-digit forces as follows: *(1)* force modulation to object weight in CTS patients would be less accurate than controls, as indicated by lower discrimination of force modulation to object weight; *(2)* larger across-trial variability and consistent use of larger digit forces than controls despite repetitive manipulation of the same object with a given weight due to inability to form accurate sensorimotor memories of manipulations from previous trials; and *(3)* CTS patients would be less skilled than controls in balancing multi-digit forces, thus leading to the production of moments on the object at lift-off that could interfere with the subsequent manipulation.

## Methods

### Ethics Statement

All participants gave their written informed consent according to the declaration of Helsinki and the protocols were approved by the Institutional Review Boards at Arizona State University and Mayo Clinic Hospital.

### Subjects

Thirteen Carpal Tunnel Syndrome (CTS) patients (2 males, 11 females) and thirteen age- and gender-matched healthy volunteers participated in this study. Subjects' average height and weight were 167.2±4.2 cm and 84.4±7.2 Kg, respectively, for CTS patients and 168.1±3.4 cm and 75.9±5.5 Kg, respectively, for controls. The gender distribution of our CTS patients reflects the higher incidence of women than men [34]–[35]. The diagnosis of CTS was based upon both clinical symptoms and confirmatory electrodiagnostic tests (Table 1; normal values are shown in Table 2). The clinical symptoms and electrodiagnostic test results were reviewed by the same neurologist (Mayo Clinic Hospital, Phoenix). All patients had symptomatic CTS at the time of testing grip function and were referred to the EMG laboratory for electrodiagnostic studies by their physician specifically to evaluate for complaints of hand paresthesias and suspected CTS. For the group, the average time from the EMG study to grip testing was 6.8 weeks. No patient received carpal tunnel steroid injection or surgical therapy prior to undergoing testing.

**Table 1:** Electrodiagnostic tests reported for patients with carpal tunnel syndrome.

No	CTS Patients									Control
	Gender	Age	Handedness	Tested hand	Electrodiagnostic test results (abnormal values in bold)^1^					Age
					Nerve Study	Amplitude^2^	Velocity^3^(m/s)	Distallatency (ms)	F-waveLatency^4^ (ms)	
1	F	48	R	L	Median sensory	**11.4**	57	**2.8**		48
					Ulnar sensory	35.4	58	1.6		
					Median motor	10	54	**5.6**	29.7	
					Ulnar motor	9.5	55	2.4	26.2	
2	M	54	R	R	Median sensory	**13.5**		**2.9**		54
					Ulnar sensory	19.3		1.7		
					Median motor	8.7	52	**6**	35.1	
3	F	57	R	R	Median sensory	71.2	59	**2.5**		59
					Ulnar sensory	27.4	60	2.3		
					Median motor	11.5	57	4.1	26.1	
					Ulnar motor	12.5	55	3.3	27.3	
4	F	60	R	R	Median sensory	**10**	62	**3.3**		60
					Ulnar sensory	19	63	1.7		
					Median motor	9.6	60	**4.8**	26.2	
					Ulnar motor	8	63	2.3	27.3	
5	F	56	R	L	Median sensory	60.2		2.3*		56
					Ulnar sensory	15.2		1.7		
					Median motor	8.7	59	3.9		
6	F	30	R	R	Median sensory	53.8	64	**2.4**		30
					Ulnar sensory	27.5	58	2		
					Median motor	11.7	59	3.4	24.1	
					Ulnar motor	13.6	65	2.4	23.1	
7	M	52	R	L	Median sensory	15.2		**4.0**		54
					Median motor	8.4		**4.8**		
					Ulnar motor	15.1	51	2.7		
8	F	56	R	R	Median sensory	**17**	53	**5.4**		56
					Ulnar sensory	24.2	74	1.7		
					Median motor	8.8		**7.1**	31	
					Ulnar motor	11	67	2.8	23.9	
9	F	42	R	R	Median sensory	**45.2**		**2.5**		40
					Ulnar sensory	26.1		1.8		
					Median motor	11.8	55	3.9		
10	F	55	R	R	Median sensory	63.5	66	**2.8**		55
					Ulnar sensory	34.8	66	1.7		
					Median motor	8.9	52	**5**	25.9	
					Ulnar motor	14.9	73	3	25.4	
11	F	48	R	R	Median sensory	51.1	62	**2.6**		47
					Ulnar sensory	44.2	62	1.8		
					Median motor	7.2	51	**5.3**	27.5	
					Ulnar motor	15.1	64	2.7	25.7	
12	F	47	R	L	Median sensory	84.6	63	**2.5**		46
					Ulnar sensory	42.5	55	2		
					Median motor	10	51	3.9	27.1	
					Ulnar motor	13.9	67	3.1	25.8	
13	F	60	R	R	Median sensory	**27.7**	59	**3.6**		59
					Ulnar sensory	27.9	61	1.8		
					Median motor	6.1	53	**5.8**	28.2	
					Ulnar motor	9.9	63	2.9	25.8	

1 Normal values are listed in Table 2. Sensory studies are orthodromic except patient 7, who had an antidromic median sensory study.

2 Amplitude values for sensory studies are microvolts and motor studies are millivolts.

3,4 Conduction velocities and F-wave latencies were normal for all nerve studies.

**Table 2:** Normal median and ulnar nerve conduction values, Mayo Clinic Arizona EMG Laboratory.

Nerve	Age < 60		Age ≥ 60^2^	
**Median**	Amplitude^1^	Wrist latency (ms)	Amplitude^1^	Wrist latency (ms)
Orthodromic sensory	≥50	<2.3	M≥17.4; F≥40.1	<2.5
Antidromic sensory	≥15	<3.5	M≥12.2; F≥15.9	<3.7
Motor	≥4	<4.5	≥ 4.5	M:<4.4; F<3.8
**Ulnar**				
Orthodromic sensory	≥15	≤ 2.3	M≥3.4 ; F≥14.4	<2.3
Antidromic sensory	≥ 10	<3.1	M≥3.9; F≥15.9	M<3.5; F<3.1
Motor	≥ 6	<3.6	≥ 4.8	M: <3.2; F<2.9

1 Amplitude values for sensory studies are microvolts and motor studies are millivolts.

2 Note that some normal values for subjects 60 years old and older are gender specific. M  =  male; F  =  female.

For inclusion in our study, CTS patients had to exhibit at minimum a prolonged median nerve distal sensory latency (antidromic or orthodromic, relative or absolute). Even though some CTS patients received sensory (Semmes-Weinstein monofilaments) and provocative tests (Durkan's nerve compression, Phalen's and Tinel's tests), the diagnosis of CTS was ultimately based on clinical symptoms and electrodiagnostic tests. Electrodiagnostic tests are considered the best available diagnostic standard for CTS [36]–[38], and thus preferable to provocative tests (for review see [39]–[40]). Eligibility for participation in our study as a control subject included absence of CTS-like symptoms and age that matched that of CTS patients within ± 2 years. This was further verified using the above sensory and provocative tests. Detailed clinical history of CTS patients and controls was carefully reviewed and we further verified eligibility for participation based on the following exclusion criteria: 1) clinical history or electrodiagnostic test results indicating ulnar, radial or proximal median neuropathy, brachial plexopathy, cervical radiculopathy or polyneuropathy, 2) orthopaedic, joint degeneration (i.e., arthritis, verified by x-ray) affecting the hand or cervical spine, 3) visual problems that would interfere with our grasp task, 4) co-existing central nervous system disease (e.g., multiple sclerosis, motor neuron disease, myasthenia gravis, Parkinson's disease, dystonia) revealed in medical history 5) significant rigidity as assessed through range of motion testing, 6) active psychiatric illness, 7) pregnancy, 8) thyroid disorders, 9) introduction of clinically significant dose change of medication known to affect motor or sensory function within 3 months of enrollment, 10) history of hand surgical interventions or corticosteroid injections for carpal tunnel syndrome and/or other musculoskeletal hand disorder, and 11) older than 60 years. Only patients with idiopathic CTS were included in the study. All CTS patients and controls were right-handed (self-reported). Only the CTS-affected hands were tested in the CTS patients. Therefore, four CTS patients were tested on their left hand and nine patients were tested on their right hand. The tested hand of control subjects was matched to the hand tested in CTS patients. All participants were naïve to the purpose of the study.

The electrodiagnostic studies confirmed the diagnosis of CTS in all patients by demonstrating prolonged latency of the median nerve localized to the wrist segment where the carpal tunnel is located (Table 1). Additionally, patients with CTS often exhibit a reduction of the sensory nerve action potential (SNAP) which is believed to correlate with loss of sensory axons. In our patients, the average SNAP amplitude was 40.3 microvolts which is below the lower limit of normal (50 microvolts). However, because of the broad range of SNAP amplitude in normal individuals, patients with mild forms of CTS may show sensory responses above the lower limit of normal. For this reason, a patient's SNAP amplitude is often compared to his/her opposite side. In this study, 7 patients had median SNAPs above the lower limit of normal. In 4 of these patients, the “normal” median SNAP on the symptomatic side was on average 35 microvolts less than the other hand. In the other 3 patients, CTS involving the other hand prevented comparison. Therefore, most of our patients (10 out of 13) had lower than normal sensory SNAP amplitudes, hence evidence of axonal loss.

To summarize, the electrodiagnostic tests used to select our CTS patients indicated abnormalities in two parameters that are considered important for grasp control: slowing of conduction velocity in sensory afferents and axonal loss. With regard to slower conduction velocity in sensory afferents, experimental and modeling work suggest that the timing patterns of action potentials from tactile afferents may play a significant role in fingertip force control, e.g., discrimination of force direction (for review see [8]). With regard to evidence of axonal loss exhibited by most of our patients, a smaller number of axons would affect the spatial resolution of tactile input of mechanoreceptors of the fingertips as well as the integration and number of inputs reaching primary sensory cortex. For brevity, we will use the term ‘sensory deficits’ to indicate the consequences of both slower conduction velocity and axonal loss of the median nerve in CTS patients.

### Apparatus

**Figure 1:**
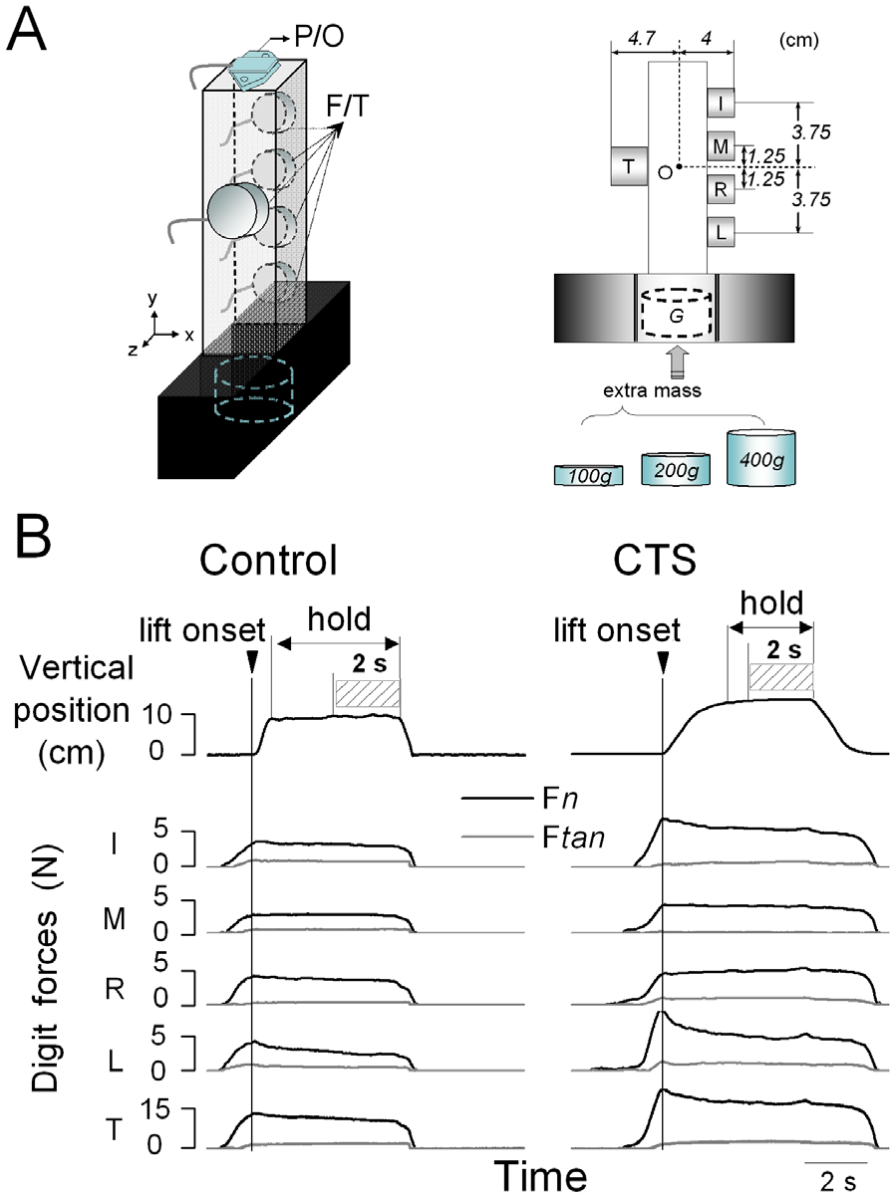
Experimental setup and variables. Panel A shows the grip device used for the experiments. Force/torque sensors (F/T) are mounted on both sides of the device to measure forces and moment of forces exerted by each digit (thumb, index, middle, ring, and little fingers: T, I, M, R, and L, respectively). A position/orientation (P/O) sensor was mounted on the top of the device to measure object kinematics. A mass (*G*: 100 g, 200 g, or 400 g) was inserted at the bottom of the grip device for each experimental condition. Dimensions are in cm. Panel B shows, from top to bottom, the time course of the object vertical position and digit normal and tangential forces (F*n* and F*tan*, respectively). Force traces are aligned with object lift onset (vertical line). Forces were analyzed at object lift onset and the last 2 seconds of object hold (striped area) used for analysis of F*n*. Data are from one representative CTS patient (S3) and her matched control (right and left column, respectively) performing the task on the third trial (445 g condition).

The grip device used for our experiments is shown in Figure 1A. Five six-component force/torque transducers (F/T, ATI Industrial Automation, Apex, NC) were used to measure three force and three moment-of-force components produced by each digit. The surface of each sensor was covered with insulating circular plastic plates of the same material (average static coefficient of friction: 0.89). The F/T sensors for the thumb (Nano-25) and each finger (Nano-17) were mounted on opposite sides of a polyvinyl chloride vertical box such that all sensors were parallel to the vertical axis of the grip device (Figure 1A). The center of the thumb sensor was aligned with the midpoint between the middle and ring finger sensors. The thumb sensor was positioned at the midpoint between the middle and ring finger to allow comparison with previous studies of whole-hand grasping [41]. The distance between the thumb and finger sensors was 8.7 cm. An electromagnetic position/orientation tracking sensor (P/O, Polhemus Fastrak, Colchester, VT; 0.075 mm and 0.05° resolution; Figure 1A) was placed on the grip device to measure the object translation and rotation. We changed object weight by inserting a mass (*G*, Fig. 1A) in the bottom box of the grip device whose center was aligned with the approximate center of gravity of the grip device (O, Figure 1A). The signals from each F/T sensor were acquired by five 12-bit A/D converter boards (National Instruments, Austin, TX) at a sampling frequency of 1 kHz. Collection of position data was triggered by the onset of force data acquisition and collected on a separate computer at a sampling frequency of 80 Hz. Force and position data were synchronized offline for analyses. Custom software (LabVIEW 6.1, National Instruments) was used to acquire, display and store force data.

### Experimental procedures

Before the experiment, subjects were asked to sit in a chair facing the grip device with the shoulder of the tested hand aligned with the grip device to ensure that the object could be comfortably grasped. Subjects were instructed to wait for a ‘go’ signal, after which they reached, grasped, lifted ∼ 10 cm from the table, held for ∼ 4 s until given a second verbal cue, and replaced the grip device on the table at a comfortable, self-selected pace. One of the experimenters visually verified that the subject contacted each sensor with the tip of a single digit. Subjects were instructed to lift and hold the grip device vertically. This is an important task requirement for testing CTS patients' ability to properly coordinate multi-digit forces (see below).

We studied the effect of CTS on multi-digit force coordination using a blocked weight presentation where a given object weight was presented over consecutive trials. Subjects were instructed to perform the above task with three different mass conditions: 445 g, 545 g, and 745 g. Each of these weight conditions was obtained by adding a mass (100, 200, or 400 g) at the bottom of the device. Subjects performed 7 consecutive lifts per weight condition, thus resulting in a total of 21 trials. Subjects were unaware of the object weight on the first trial, but were aware that it would remain the same within the block of 7 trials. The three weight conditions were presented in a counterbalanced order across CTS patients, and in the same order between each CTS patient and his/her matched control. Subjects were given a 10-s rest period between trials and experimental conditions. The entire experiment lasted about twenty minutes to prevent pain, fatigue, or worsening of the CTS symptoms. None of our subjects reported any of these adverse reactions.

### Data processing

Force and position data were temporally aligned offline by re-sampling the position data through linear interpolation at the same frequency of the force data (1 kHz). Analyses were performed using MATLAB, Excel, and SPSS software. Fig. 1B shows kinetic and kinematic data from a CTS patient and her control. Object *lift onset* and object *hold* were used to define task epochs within which forces were analyzed. Object lift onset was defined as the time at which the vertical position of the grip device crossed and remained above a threshold (mean + 2 SD of the signal baseline) for 200 ms (Fig. 1B). The end of object lift onset was defined as the instant at which the absolute derivative of the object vertical position dropped less than 3% of its maximal value during object lift. Object hold was defined as the time period between the end of object lift and the onset of the object downward movement which was defined as the instant at which the absolute derivative of the object vertical position increased more than 3% of its maximal value during the object downward movement. Object lift onset was used to examine anticipatory scaling of digit forces (timing of peak force rates relative to object lift onset; see below) to object weight based on previous manipulations, whereas object hold was used to evaluate subjects' ability to adapt digit forces as a result of sensory feedback acquired following object lift onset. As force transients occur at the onset and shortly after the end of object hold, experimental variables related to object hold were analyzed by averaging over the last 2 s of the steady portion (striped box, Fig. 1B).

1. **Digit forces.** Digit *tangential force* (F*tan*) is the vertical force component parallel to the grip surface produced by each digit to lift the object (Fig. 1B). Digit *normal force* (F*n*) is the force component perpendicular to the grip surface (Fig. 1B). We processed digit forces as follows: *(a)* sum of F*n* and F*tan* exerted by all digits (F*_G_* and F*_T_*, respectively); *(b)* difference between F*_G_* at object lift onset and F*_G_* during object hold (ΔF*_G_*); *(c) peak* F*_G_* and F*_T_ rate* computed within the period from contact of the first digit (signaled by F*n* crossing and remaining above a threshold of 5 standard deviations of the mean baseline force for 300 ms) and end of object lift; *(d) time of peak* F*_G_* and F*_T_ rate* relative to object lift onset; *(e) load phase* defined as the time between onset of load force and object lift onset (onset of load force was signaled by F*tan* crossing and remaining above a threshold of 5 standard deviations of the mean baseline for 300 ms); *(f)* F*_G_* across-trial variability defined as the standard deviation of mean F*_G_* averaged across trials 2 through 7 (we omitted trial 1 because all subjects produced much larger forces than on subsequent trials due to their lack of knowledge of object weight); *(g)* F*_G_* within-trial variability defined as the standard deviation of F*_G_* over the last 2 s object hold and normalized by average F*_G_*, i.e., the coefficient of variation; and *(g)* F*n* exerted by each finger expressed as percentage of thumb F*n* (normal force sharing pattern, SF*n*).
2. **Moment of forces.** Analysis of moment of force focused on moments exerted in the frontal plane (*yz* plane) about the origin ‘O’ (the approximate object's center of gravity; Fig. 1A) at object lift onset. The rationale for this analysis is that it provides further insight into subjects' ability to coordinate multi-digit forces. Specifically, our task requires subjects to coordinate multi-digit forces such that at object lift onset the object is lifted vertically, hence that *(a)* the sum of all fingers normal forces match thumb normal force, *(b)* the sum of all finger tangential forces match thumb tangential force, and *(c)* the sum of moments produced by F*n* and F*tan* is zero, i.e., the net moment (M*net*) exerted on the object is zero. Conversely, deviations from zero net moments would denote subjects' inability to coordinate the partitioning of forces exerted by the thumb relative to forces exerted by all fingers.
  M*net* produced on the grip device consists of two moment components: digit *normal moment* (M*n*) and digit *tangential moment* (M*tan*). Our task requires F*n* generated by the thumb (F*n*_T_) and by its opposing fingers (F*n*_IMRL_) to be equal but opposite to each other, therefore M*n* can be calculated as:
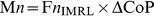
(1)where ΔCoP is the vertical distance between the center of pressure of the thumb and fingers [41]. M*tan* is the moment of tangential forces produced by the thumb (F*tan*_T_) and by its opposing fingers (F*tan*_IMRL_) about ‘O’:
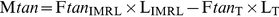
(2)where L_IMRL_ and L_T_ denote lever arm of F*tan* at the fingers and thumb, respectively.
3. **Peak object roll.** Peak roll was used to further quantify the behavioral consequences of multi-digit force coordination implemented at object lift onset and throughout the lift [42]–[43].

All of the above variables are standard metrics to characterize force coordination and/or behavioral consequences (object roll) used by studies of precision grip [5], [30], [44] and whole-hand grasping [45]–[47].

### Statistical analysis

To determine differences between CTS and controls in the multi-digit force modulation to object weight we performed analysis of variance (ANOVA) with repeated measures on *(a)* peak object roll, *(b)* F*_G_* at object lift onset and hold, *(c)* within-trial (object hold) variability of F*_G_*, and *(d)* M*net*, M*n*, and M*tan* object lift onset, with *Weight* (three levels: 445, 545, and 745 g) and *Trial* (seven levels: 1^st^ through 7^th^ trial) as within-subject factors, and *Group* (two levels: CTS and controls) as between-subject factor. Linear regression analysis was performed on M*n* vs. M*tan* at object lift onset for all trials pooled across weight conditions for each subject to quantify the coordination between the two moment components. Negative correlations between M*n* and M*tan* denote error compensation acting to minimize across-trial variability in the sign and magnitude of the net digit moment [48]. The Pearson's correlation coefficient (*r*) was z-normalized before averaging across subjects within each group and paired t-test was used to assess group differences in the *r*-value and intercept of the linear fits.

For the following analyses, we computed the mean and variability of digit forces from trial 2 through 7. To quantify the group differences in the adaptation of digit force sharing patterns (SF*n*) to object weight, we performed separate ANOVAs with repeated measures with *Weight*, *Finger* (four levels: index, middle, ring, and little finger) as within-subject factors, and *Group* as the between-subject factor on SF*n* at object lift onset and hold. We also used ANOVA with repeated measures with *Weight* and *Phase* (two levels: object lift onset, object hold) as within-subject factors and Group as the between-subject factor on F*_G_*. Fisher's z-transformation was performed on SF*n* before performing statistical analysis. To determine the consistency with which subjects coordinated multi-digit forces, we performed separate ANOVAs with repeated measures with *Weight* as the within-subject factor, and *Group* as the between-subject factor, on the across-trial variability of F*_G_* at object lift onset and during object hold. To quantify group differences in the within-trial temporal evolution of multi-digit forces, we performed paired t-tests on the average difference between forces at object lift onset and hold (ΔF*_G_*). For repeated measures analysis, Mauchly's test was used to test for sphericity. Greenhouse-Geisser correction was used when the sphericity assumption was violated. When appropriate, we performed post hoc comparisons with Bonferroni adjustments. A significance level of 0.05 was used for all comparisons.

## Results

Both CTS and control subjects successfully completed the experiment for each weight condition as instructed without slipping, dropping, or significantly tilting the object. Patients did not exhibit qualitative differences in how they grasped or lifted the object, and none reported having difficulty with executing the task.

### Task performance

Subjects were asked to lift the object while keeping the object aligned vertically. On the first trial, subjects have not experienced the object weight. Therefore, a larger object roll occurred on the first trial (1.8°±0.2°; main effect of Trial: F_[6,144]_ = 7.244; *P*<0.001) than the subsequent trials. Even though larger weights tended to cause greater peak object rolls (significant main effect of Weight: F[_2, 48_]  = 4.833; *P*<0.05; 545 g>445 g), no group difference was observed (F[_1, 24_]  = 0.16; *P*>0.05).

### Multi-digit force coordination during object hold

**Figure 2:**
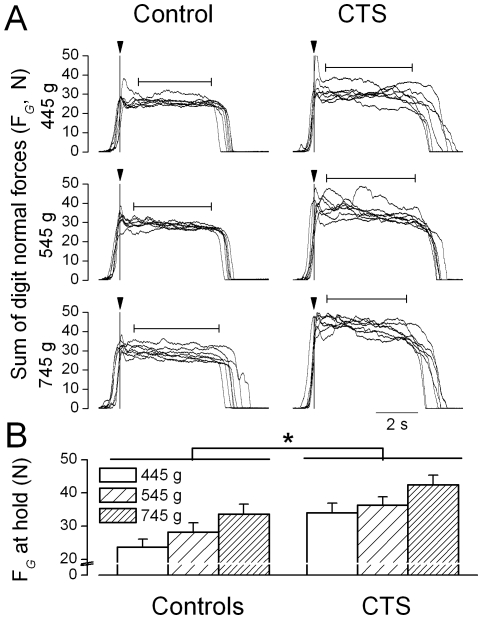
Grip forces during object hold. Panel A shows the time courses of grip force (sum of all digit normal forces, F*_G_*) from a representative CTS patient (S7) and his matched control (right and left column, respectively) across a block of trials (*n* = 7) for each weight condition. Data are aligned relative to object lift onset (vertical line). The horizontal arrows denote the mean duration of object hold averaged across trials. Panel B shows F*_G_* during object hold averaged across trials and subjects for the CTS and control group for each weight condition. Vertical error bars denote standard errors. Asterisk indicates statistically significant difference between the two subject groups (*P*<0.05).

Figure 2A shows grip force (F*_G_*) from seven trials for each weight condition performed by a representative CTS patient and his control. Although both subjects increased F*_G_* as a function of object weight during object hold, the CTS patient exerted larger F*_G_* than his control for all weight conditions. Both subject groups (Fig. 2B) increased F*_G_* during object hold with increasing object weight (controls: 23.7±2.4 N, 28.2±2.8 N, and 33.7±2.9 N; CTS patients: 34±2.9 N, 36.3±2.5 N, and 42.4±3 N, respectively; main effect of Weight: F[_2, 48_]  = 82.119; *P*<0.001). However, post hoc tests showed that significant force adaption in CTS patients occurred only when comparing the 745 g condition vs. lighter weight conditions. In contrast, controls showed significant force modulation for all pairwise weight comparisons. CTS patients exerted at least 8 N larger F*_G_* during object hold than controls at all weight conditions (main effect of Group: F[_1, 24_]  = 5.568; *P*<0.05).

**Figure 3:**
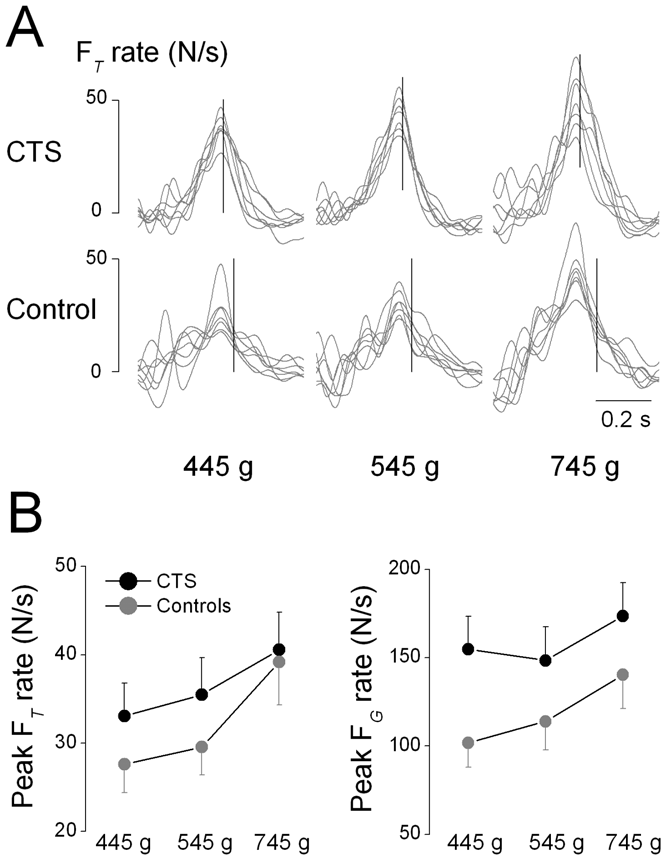
Digit force rates. Panel A shows the time course of the rate of the sum of digit tangential forces exerted by all digits (F*_T_)* rate from trial 1 through 7 for one CTS patient and her control aligned with respect to peak F*tan* rate. The vertical line denotes the time of object lift onset averaged across trials. Panel B shows peak rate of F*_T_* and F*_G_* averaged across trials 2 through 7 and subjects for each group and object weight. Vertical error bars denote standard errors.

The above scaling of F*n* as a function of object weight in CTS patients could have been due to subjects gradually increasing F*n* together with F*tan* until the object could be lifted, e.g., a ‘probing’ strategy (Gordon et al. 1993). However, even though load phase duration increased with object weight (F[_2, 48_]  = 9.022; *P*<0.01), no group differences or interactions with object weight were found (F[_1, 24_]  = 0.389; *P*>0.05). Therefore, the timing of force development prior to object lift onset was similar in both subject groups. To further explore the mechanisms of force modulation to object weight, we examined the modulation of the peak rate of the sum of all digit F*tan* (F*_T_*) to object weight and its timing relative to object lift onset. Figure 3A shows the time course of F*_T_* rate across object weights for one representative CTS patient and her control. For these two subjects, peak F*_T_* rate increased with increasing object weight and occurred shortly before or at object lift onset. Scaling of peak F*_T_* rate with object weight is considered evidence for anticipatory force modulation (Gordon et al. 1993) and was found in both CTS patients and controls (Fig. 3B, left plot; main effect of object weight: F[_2, 48_]  = 23.712, *P*<0.001; no main effect of Group; F[_1, 24_]  = 0.639; *P*>0.05). We also found that peak F*_T_* rate occurred before object lift onset in the majority of trials across all object weights in CTS patients and controls (77% and 81%, respectively).

Peak F*_G_* rate also increased with object weight in both groups (Fig. 3B, right plot; main effect of object weight: F[_2, 48_]  = 21.853, *P*<0.0001). However, the extent of peak F*_G_* rate modulation to object weight was higher in controls than CTS patients (significant interaction Mass × Group: F[_2, 48_]  = 3.602, *P*<0.05; post hocs: controls, 745 g>545 g and 445 g; CTS: 745 g>545 g). Peak F*_G_* rate occurred ∼200-300 ms before object lift onset in both groups (no main effect of Group, F[_1, 24_]  = 0.25; *P*>0.05) and relative time to lift onset tended to increase with object weight (main effect of Weight: F[_2, 48_]  = 6.267, *P*<0.005). We also found a main effect of Trial (F_[6,144]_  = 6.232, *P*<0.001), time to peak F*_G_* rate being longer on the 1^st^ vs. the rest of the trials.

### Temporal evolution of multi-digit forces across and within trials

No significant trial-to-trial changes in F*_G_* during object hold occurred in either subject group (F_[6,144]_  = 1.178; *P*>0.05). However, F*_G_* at object lift onset varied significantly from trial to trial (main effect of Trial: F_[6,144]_  = 8.941; *P*<0.001; F*_G_* on trial 1 > trial 2-7), as subjects in both groups adopted a ‘default’ grip force at object lift onset on the first trial only across all weight conditions (37.3±4.1 N and 47.9±4 N for controls and CTS patients, respectively).

**Figure 4:**
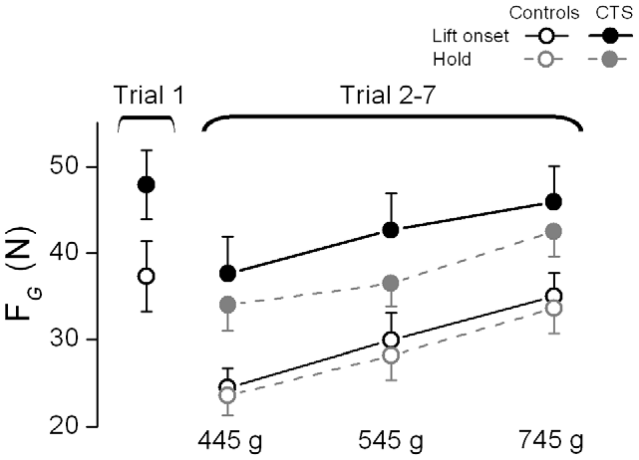
Grip force at object lift onset and object hold. Grip force (F*_G_*) at object lift onset and during object hold on the 1^st^ trial (averaged across all weights), and averaged across trials 2 through 7 are shown for the CTS and control groups (filled and open symbols, respectively) and each weight condition. Note that F*_G_* during object hold on the first trial is not plotted since F*_G_* did not change significantly across trials, i.e., F*_G_* during hold on the first trial  =  F*_G_* on trials 2-7. Vertical error bars denote standard errors.

Figure 4 shows F*_G_* produced at object lift onset on the first trial together with F*_G_* produced at object lift onset and hold averaged from trial 2 through 7 for CTS and control subjects. Both groups modulated F*_G_* at object lift onset according to object weight (main effect of Weight: F[_2, 48_]  = 51. 65; *P*<0.001) on all but the first trial (significant interaction Mass × Trial: F_[12,288]_  = 3.516; *P*<0.001). After the first trial, F*_G_* at object lift onset in control subjects was of similar magnitude to that exerted during object hold, indicating that force scaling had occurred *prior* to lifting the object and that no further modulation occurred after object lift. Subjects exerted smaller F*_G_* during object hold than at object lift onset, indicating that further digit force modulation occurred throughout object lift and the early part of object hold (main effect of Phase: F[_1, 24_]  = 5.085; *P*<0.05). We also found that CTS patients exerted larger F*_G_* than controls across all trials and weight conditions (main effect of Group: F[_1, 24_]  = 6.483; *P*<0.05). We noted that even after the object was lifted, CTS patients still exerted larger F*_G_* than controls (Figs. 2B and 4). Group differences in F*_G_* modulation at object lift onset vs. hold were statistically significant, ΔF*_G_* being larger in CTS than control subjects (4.86±2.02 N and 1.35±1.87 N, respectively; t-value = 2.25, *P*<0.05). Therefore, even though CTS patients could anticipate object weight similarly to controls prior to object lift onset (see previous section; Fig. 3), they consistently exerted larger forces and particularly so when they were about to lift the object.

### Within and across-trial force variability

**Figure 5:**
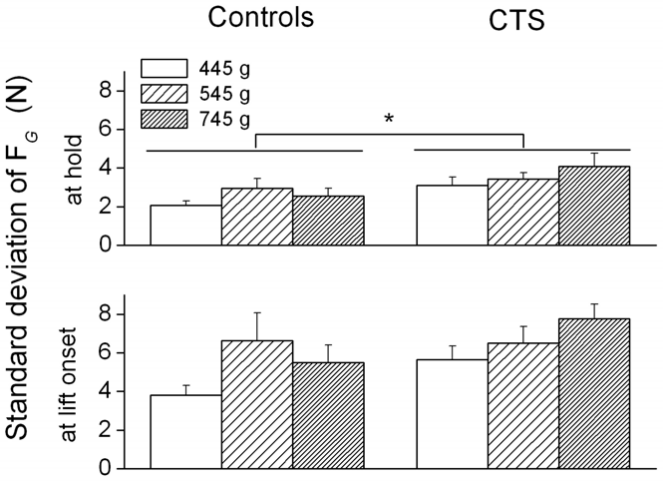
Across-trial variability of grip force at object lift onset and hold. The standard deviation of grip force (F*_G_*) at object lift onset and during object hold averaged across trial 2 through 7 is shown for controls and CTS patients (left and right column, respectively) and for each weight condition. Vertical error bars denote standard errors.

After the first object lift, both groups learned to minimize object roll and modulate F*_G_* to object weight in an anticipatory fashion (at object lift onset) and during object hold. Within-trial variability of F*_G_* during object hold were similar across groups (F[_1, 24_]  = 0.009; *P*>0.05). CTS and controls exhibited higher across-trial variability in F*_G_* at object lift onset than object hold (t-value  =  5.86; *P*<0.001; Fig. 5, bottom plots). However, CTS patients were significantly more variable across trials than controls during object hold (main effect of Group: F[_1, 24_]  = 5.556; *P*<0.05; Figs. 2A, 5, top plots).

### Multi-digit force sharing patterns

**Figure 6:**
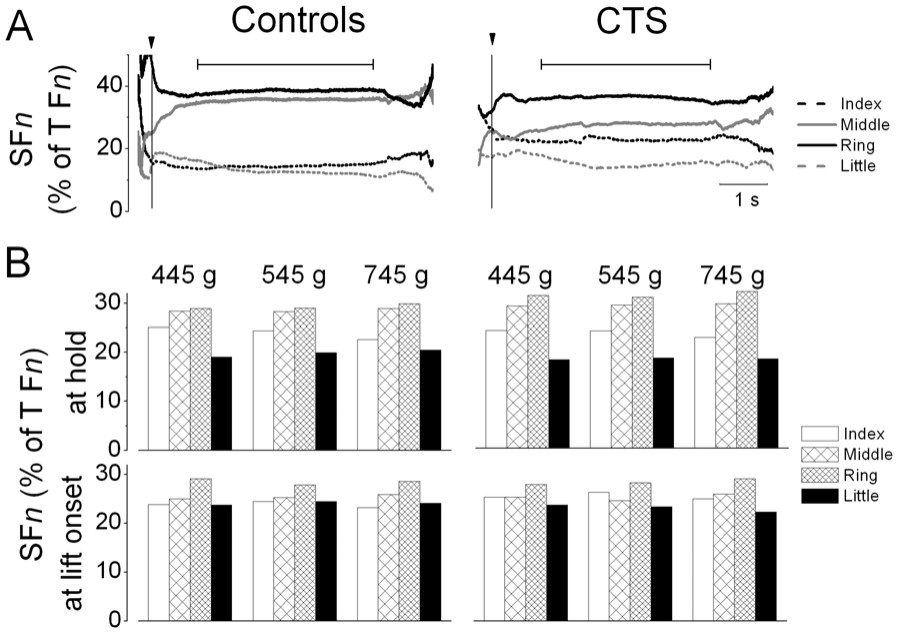
Digit force sharing patterns. Panel A shows the time course of individual finger normal forces expressed as percentage of thumb (T) normal force from a representative CTS patient (S1) and her control (5^th^ trial, 445 g condition). Data are aligned relative to object lift onset (vertical line). The horizontal bars denote object hold. Panel B shows force sharing patterns (SF*n*) averaged from trial 2 through 7 and all subjects for each weight condition measured at object lift onset and hold.

Figure 6 shows finger forces normalized by thumb normal force (SF*n*) as a function of time for two representative subjects (A) and averaged across all subjects (B). The control and CTS patient shown in Fig. 6A exhibited fairly stable SF*n* shortly after object lift onset (vertical line), with the middle and ring finger exerting the largest share of thumb F*n*. However, these two subjects also exhibited small differences in how they shared finger forces relative to thumb F*n*. Across all subjects, the middle and ring fingers combined exerted over 60% of thumb F*n* (main effect of Finger only: F_[3,72]_  = 23.597; *P*<0.001 and F_[3,72]_  = 4.448; *P*<0.01 for object hold and object lift onset respectively). Importantly, no group differences were found in force sharing patterns either at object lift onset (F[_1, 24_]  = 1.91; *P*>0.05) or during object hold (F[_1, 24_]  = 2.944; *P*>0.05).

### Coordination between components of moments of force

To prevent large object roll during object lift subjects have to coordinate digit normal and tangential forces at object lift onset such that the net moment (M*net*) is equal to zero. However, if a non-zero M*net* is generated at object lift onset, subjects can generate corrective force responses during the lift but only at reaction time latencies. We found that both subject groups exerted non-zero M*net* of force at object lift onset, but improved with practice as smaller M*net* were exerted after the first two trials (significant main effect of Trial: F_[6,144]_  = 4.05; *P*<0.005). However, CTS patients were farther away from the optimal zero M*net* strategy as they exerted significantly larger net moments at object lift onset relative to controls (CTS patients: 4.79±0.46 N•cm; controls: 3.23±0.46 N•cm; significant main effect of Group: F[_1, 24_]  = 5.683; *P*<0.05). Further analysis revealed that this group difference was caused by CTS patients exerting a significantly larger M*tan* (main effect of Group: F[_1, 24_]  = 6.026, *P*<0.05) but similar M*n*. We found that CTS patients used a significantly different tangential force sharing pattern relative to controls (significant interaction *Digit × Group;* F_[4,96]_  = 2.671, *P*<0.05). Post hoc tests revealed that this interaction was caused by CTS patients exerting F*tan* in the downward direction with the index finger relative to the rest of the digits, whereas controls exerted F*tan* in the upward direction with all digits (*P*<0.05).

**Figure 7:**
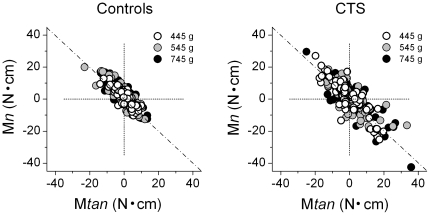
Coordination between normal and tangential moments. Normal moment (M*n*) at object lift onset is plotted against tangential moment (M*tan*) at object lift onset. Data are from all trials, weight conditions, and subjects from each group. The diagonal line denotes the range of available solutions that result in zero net moment.

One way of generating a zero M*net* of force at object lift onset is to generate zero normal and tangential moments (M*n* and M*tan*, respectively), i.e., symmetrical F*tan* and collinear F*n*. However, if one of these moment components is non-zero (say, M*tan*), a zero M*net* can still be generated but only if the other moment component (M*n*) covaries negatively. To determine the extent to which the two subject groups differed in their ability to coordinate M*n* and M*tan*, we performed linear regression between M*n* and M*tan* for each subject and across all weight conditions. Figure 7 shows the scatter plots for CTS patients and controls based on data from individual trials from each subject and weight condition. Surprisingly, both groups showed negative covariations between the two moment components that were characterized by similar correlation coefficients (CTS: 0.87±0.02; controls: 0.83±0.03). Furthermore, the center of the data distribution is close to zero intercept, indicating that the covariation between M*n* and M*tan* acted to minimize across-trial deviations from the required zero net moment. Note, however, that the plots in Fig. 7 also reveal a larger scatter in the M*n* vs. M*tan* relations from CTS patients than controls, part of which reflects their larger across-trial variability in F*_G_* (described above). Lastly, even though both subject groups implemented a negative covariation between the two moment components, this coordination was more effective in controls as indicated by the intercept of the linear fit being significantly closer to zero than in CTS patients (2.48±0.41 N and 4.23±0.7 N, respectively; t-value  =  2.12, *P*<0.05).

## Discussion

Controls and CTS patients were able to grasp, lift, hold, and replace objects of different weights by modulating digit forces accordingly, even though CTS patients exerted larger digit forces than controls. Besides this observation, a more detailed analysis revealed differences in multi-digit force coordination relative to controls: *(a)* digit force modulation to object weight by CTS patients did not discriminate lighter weights as accurately as controls; *(b)* CTS patients exhibited a larger across-trial variability in digit force control; *(c)* unlike controls, after the first trial CTS patients consistently exerted larger forces at lift onset than during object hold; and *(d)* CTS patients were less able to balance digit forces than controls, resulting in unnecessary net moments at object lift onset. These results are discussed in the context of behavioral consequences of chronic median nerve compression on dexterous coordination of multi-digit forces for whole-hand grasping.

### Modulation of multi-digit forces to object weight

During multi-digit prehension tasks, healthy adults share total F*_G_* among thumb and fingers in a similar way across object weights [49]. The present results are consistent with this observation indicating that both controls and CTS patients used object weight-independent force sharing patterns that were maintained from object lift onset through object hold (Fig. 6). Thus, despite the excessive F*_G_* used by CTS patients, the ability to proportionally scale individual digit normal forces indicates an intact ability to coordinate multi-digit forces. The fact that the object did not move laterally nor was tilted during the lift indicates that patients were able to attain normal force equilibrium among CTS-affected and non-affected digits. Based on evidence from studies of two-digit grasping in CTS patients [29], we speculate that CTS-affected digits exerted excessive forces. The task requirement of attaining equilibrium of normal forces among all digits would then result in the compensatory strategy of exerting excessive forces also with CTS non-affected digits despite their intact sensorimotor capabilities.

CTS patients were still able to scale multi-digit F*_G_* in an anticipatory fashion to object weight (Fig. 4), thus suggesting a residual ability to process sensory feedback, form sensorimotor memories, and use them to modulate digit forces prior to object lift onset. Given the sensory deficits identified by electrodiagnostic tests (Table 1), possible explanations for this residual ability to modulate F*_G_* to object weight are that spared somatosensory feedback from the hand and/or that more proximal sources of feedback were also used. Specifically, it is possible that feedback from muscle, joint, and tendon mechanoreceptors in the forearm and upper arm – whose function is spared by median nerve compression – could have been integrated with residual somatosensory feedback from the hand to infer object weight after the first object lift.

However, we also found evidence indicating that force modulation to object weight was less accurate in CTS than controls. Unlike controls, F*_G_* and peak F*_G_* rate did not discriminate between the lighter object weights (445 vs. 545 g), but only between these and the heaviest object weight (745 g). The more similar peak force rates across object weights during the load phase suggest a sustained larger force compared to controls, which ensures higher F*_G_* at object lift onset to prevent potential object slips (see below). This between-group difference in the lower discrimination of force modulation to object weight emphasizes the role of somatosensory feedback from hand mechanoreceptors for fine control of hand muscles and digit forces. This further indicates that behavioral deficits in CTS patients might be particularly pronounced in tasks that require fine regulation of fingertip forces for manipulating light or fragile objects.

### CTS and use of excessive digit forces

Exertion of significantly larger grip forces (F*_G_*) by CTS patients than controls was found at object lift onset *and* during object hold regardless of object weight (Fig. 3). Specifically, even though CTS patient reduced grip forces after manipulating the object on the first trial, unlike controls CTS patients *continued* to exert excessively large F*_G_* across consecutive trials of each object weight condition despite lifting the same object for several consecutive trials (Fig. 2A). This behavior may result from an inability to acquire and process somatosensory feedback (see Introduction for definition of ‘somatosensory feedback’ in the context of hand control in CTS) from previous trials and use it for accurate scaling digit forces on subsequent lifts. This interpretation is consistent with the results of electrodiagnostic tests revealing that most of our CTS patients had pure or predominant sensory axon loss with no or minimal motor axon loss (Table 1). Therefore, the significantly larger F*_G_* force in CTS patients is likely to reflect a chain reaction that starts with *(a)* the inability to acquire accurate feedback from CTS-affected digits throughout grasping and manipulation which, in turn, leads to *(b)* storage of inaccurate sensorimotor memories linking object weight with the forces necessary to efficiently manipulate the object, and ultimately *(c)* prevents patients from adjusting multi-digit forces to object weight on subsequent trials to the same level of accuracy as controls.

The finding of excessively large F*_G_* is consistent with one study of manipulation in individuals with chronic or acute median nerve compression. Lowe and Freivalds [29] reported that CTS patients used significantly larger normal forces than necessary to prevent object slip during tool manipulation with the thumb and index finger. Similar findings have been reported by studies of healthy subjects using mechanical compression of the median nerve [20] and injection of anesthesia into the carpal tunnel [50] or digits [30]–[33], [51]–[53]. The interpretation of these findings is that excessive F*_G_* represents an attempt to compensate for the loss of tactile feedback, thus minimizing the risk of object slip [20]. Our data further suggest that the implementation of this compensatory strategy was nevertheless characterized by significantly larger across-trial variability relative to controls, further underscoring the role of tactile feedback for producing multi-digit forces in a consistent and efficient fashion. Note that other studies have reported similar grip force modulation in CTS patients and controls as a function of texture [23] and during point-to-point arm movements with a hand-held instrumented object [22]. However, the discrepancy between the results of these studies and those reporting the use of larger grip forces than controls ([29], present results) is difficult to interpret due to methodological and task differences, e.g., pooling CTS patients with patients affected by axonal or demyelinating sensory polyneuropathy [22].

### Effects of CTS on grasp planning and execution

Analyses of trial-to-trial force adaptation to a given object weight, as well as across object weights, provided further insights into CTS-induced sensorimotor deficits underlying hand control. Specifically, after experiencing object weight on the first trial, both groups used smaller F*_G_* at lift onset and adapted them to the object weight by using sensorimotor memories of the previous lift (Fig. 3) [30], [44]. CTS patients' ability to modulate F*_G_* to object weight points to a residual ability to utilize somatosensory feedback to plan multi-digit forces (see above). However, an important difference between CTS patients and controls was that the latter group anticipated F*_G_* used to hold the object before lifting the object, whereas CTS patients further decreased F*_G_* after object lift. This suggests that, unlike controls, CTS patients *consistently* overshot, from the second trial onwards, F*_G_* before lifting the object. We rule out the possibility that this consistent overshoot was due to CTS patients having problems with timing the development of F*_G_* before object lift onset because time to peak F*_G_* rate was statistically indistinguishable from controls. Therefore, we offer two alternative interpretations of this finding. One interpretation is that the decrease in F*_G_* following object lift could have been due to CTS patients sensing, throughout object lift and in the early part of the hold phase, that F*_G_* were larger than necessary to prevent object slip and modulated them accordingly during object hold. However, this interpretation raises the question of why, if CTS patients were able to sense the mismatch between actual and required F*_G_*, they continued to overshoot F*_G_* prior to object lift on each of trials 2 through 7, and not properly plan F*_G_* at lift onset. A non-mutually exclusive and more likely interpretation is that the consistently larger F*_G_* at object lift onset than during the static phase is a strategy learned during every-day activity to compensate for the deficit in tactile feedback signaling distinct events of the manipulation, e.g., force development prior to object lift, the dynamic force modulation during object lift, and isometric force generation during object hold. Specifically, CTS patients may prefer to use an extra safety margin of grip forces particularly during the dynamic phase for which the necessary digit forces might be more challenging to accurately anticipate than during the static hold phase. Given the above-described CTS patients' decreased ability to discriminate object weights and larger across-trial force variability, we propose that the consistently larger forces at object lift onset vs. object hold represent a compensatory strategy that reflects different requirements and challenges to grasp stability associated with dynamic vs. static phases of manipulation.

### Loss of dexterity in multi-digit force coordination in CTS

Our task required subjects to distribute forces among the digits such that no net normal force or moment would be generated during object lift and hold. When using a whole-hand grasp, production of a net zero moment on the object can be attained through an infinite number of solutions to cancel out the moment generated by F*_G_* (M*n*) with that generated by tangential forces (M*tan*) [48]. CTS patients and controls were able to coordinate M*n* and M*tan* as required by the task, i.e., by implementing a negative covariation between the two moment components. However, CTS patients exhibited a significantly larger non-zero net moment than controls at object lift onset, thus indicating a lower ability to coordinate grip and tangential forces, as well as significantly different tangential force sharing patterns relative to controls. Importantly, our findings suggest that this grasp execution ‘error’ would occur *every time* that an object is lifted.

The generation of larger moments on the object at lift onset could result from the interaction of several factors. For example, the inability to accurately integrate somatosensory feedback from each digit would interfere with the spatial and temporal coordination of hand muscle activity to balance the two moments. Furthermore, intrinsic hand muscles, some of which may be affected in CTS, play an important role in fine regulation of moments of force [54].

In conclusion, CTS does not affect macroscopic features of grasp control when adapting multi-digit forces to object weight and we propose that such modulation is mediated by proprioceptive inputs spared by CTS. However, relative to controls, multi-digit force coordination in CTS patients was characterized by the consistent use of excessively large digit forces despite repeated exposure to manipulating the same object weight, higher across-trial digit force variability, lower discrimination of force modulation to lighter object weights, and a lower ability to minimize net moments on the object at lift onset. We interpret these behavioral deficits as resulting from impaired nerve function (slowing of sensory nerve conduction velocity and axonal loss). Such impairment may account for patients' reduced ability to form accurate sensorimotor memories of previous manipulations, or represent learned compensatory strategy to maximize grasp stability. Further studies are needed to distinguish between these two interpretations and quantify the behavioral effects of CTS on fine manipulation.
